# Inclusion of Fructooligosaccharide and Resistant Maltodextrin in High Fat Diets Promotes Simultaneous Improvements on Body Fat Reduction and Fecal Parameters

**DOI:** 10.3390/molecules23092169

**Published:** 2018-08-28

**Authors:** Wei-Min Kao, Chih-Ren Chang, Tsai-Ju Chang, Shang-Yan Li, Wei-Jen Chen, Chi-Fai Chau

**Affiliations:** 1 Department of Food Science and Biotechnology, National Chung Hsing University, 145 Xingda Road, Taichung 40227, Taiwan; d9643202@mail.nchu.edu.tw (W.-M.K.); d104043001@mail.nchu.edu.tw (C.-R.C.); d106043002@mail.nchu.edu.tw (T.-J.C.); g105043317@mail.nchu.edu.tw (S.-Y.L.); 2 Syngen Biotech Co., Ltd., Building A, No. 154, Kaiyuan Rd., Sinying, Tainan 73055, Taiwan; chen.william@standard.com.tw

**Keywords:** body fat, fecal bacteria, fecal parameters, fructooligosaccharide, resistant maltodextrin

## Abstract

This study investigated the effects of incorporating a mixture of fructooligosaccharide (FOS) and resistant maltodextrin (RMD) at a ratio of 1:2 on body fat accumulation and fecal bacterial parameters in rats. Our results indicated that high dietary fat consumption might effectively (*p* < 0.05) increase body fat, but consequently inducing a significantly (*p* < 0.05) higher growth of *C. perfringens* and retarded growth (*p* < 0.05) of the *Bifidobacterium* spp. in the large intestine. As compared with the high fat control, an incorporation of the FOS and RMD mixture at a high dose (0.97 and 1.94 g/kg body weight, respectively) could result in a significant (*p* < 0.05) reduction in feed efficiency (−16%), total visceral fat (−17.4%), non-visceral fat levels (−20.3%), and total body fat (−19.2%). Furthermore, feeding the FOS and RMD mixture at a high dose was capable to counter the above undesirable impacts by reducing the *C. perfringens* count (−14.8%) and increasing the total *Bifidobacterium* count (134.4%) and total fecal short chain fatty acids (195.4%). A supplementation of adequate amount of FOS and RMD might confer a concreted solution to the obesity and deteriorated fecal bacteria profiles due to high fat consumption.

## 1. Introduction

Obesity is one of the major current public health problems owing to lifestyle changes and an increase in hypercaloric diet availability. An imbalance between energy intake and energy expenditure can engender obesity and its associated comorbidities including diabetes, insulin resistance, and cardiovascular diseases. Deterioration in gut bacterial profiles was associated with high-fat dietary patterns, resulting in the occurrence and development of gastrointestinal diseases [1,2].

Many interventional studies have suggested that adequate consumption of dietary fibers, such as fructooligosaccharide (FOS) and resistant maltodextrin (RMD), could reduce body fat accumulation and body weight [3], and also help improve bowel functions and intestinal health [4,5]. The beneficial physiological functions of FOS and RMD included protection against colorectal diseases, immunomodulatory effect, management of diabetes mellitus and obesity, and improvement of serum lipids’ concentrations and mineral absorption [6,7,8]. A sufficient intake of FOS and RMD, as prebiotics, might also improve stool quality (e.g., pH, SCFA, frequency, microflora, and consistency), lower the risks of gastroenteritis and infections, and maintain general well-being [9,10,11].

The combination of FOS and RMD at a ratio of 3:7 has been reported to show a satisfied hypolipidemic potential [12]. Our previous in vivo preliminary study [13] also demonstrated that rats fed a mixture of FOS and RMD (namely FOS-RMD mixture) at a ratio of 1:2 (FOS 0.48 g/kg bw and RMD 0.97 g/kg bw) for eight weeks was effective in lowering body fat accumulation.

Based on the associations between high fat dietary pattern and deterioration of certain fecal parameters, as well as the ability of dietary fibers in reducing body fat accumulation and promoting probiotic growth, it was believed that the administration of FOS and RMD might confer a concreted solution to the obesity and deteriorated fecal bacteria profiles resulted from high fat consumption. In the present study, animals were fed high-fat diets with an inclusion of a mixture of FOS and RMD. The changes in body fat accumulation, intestinal microbial growth, and different fecal parameters were determined and compared.

## 2. Results and Discussion

A summary of the total food intake, body weight, total energy intake, and feed efficiency ratio is shown in Table 1. The total food intake, which was a sum of the total amount of base diet and sample given, in the normal control (1915.9 g) was significantly (*p* < 0.05) higher than those of high fat (HF) (1624.6 g), HF1X (1640.6 g), and HF2X (1651.3 g) groups. No apparent differences in food intake among the three diet groups (HF, HFX1, and HFX2) were observed. Although the normal control (NC) group had the most total food intake, it had significantly (*p* < 0.05) lower total energy intakes (6418.3 kcal) than those of the animals fed the three high fat diets (6891.9–6969.5 kcal).

After the acclimation period, the rats’ initial weights did not differ among the four diet groups. As for the weight changes, all three high fat groups (262.3–302.0 g) presented a markedly (*p* < 0.05) higher weight gain than the normal diet group (199.5 g), indicating the effectiveness of high fat diet in inducing obesity. The inclusion of the FOS-RMD sample into the high fat diets resulted in the least (*p* < 0.05) weight gain for the HF2X group (262.3 g). A negative association between the weight gain and the amount of sample intake was observed in Table 1. Adequate intake of dietary fibers, such as FOS and RMD, has been reported to be capable of suppressing the weight increase even with high dietary energy consumption [14,15].

As compared with the feed efficiency of the NC group, the results revealed that the high fat dietary formula used in this study would render a significant (*p* < 0.05) increase in feed efficiency up to 152–181% higher. An incorporation of the FOS-RMD mixture at a high dose (HF2X) into the high fat diet would in turn lead to a remarkable (*p* < 0.05) decrease in feed efficiency (−16.0%) versus the HF group.

A comparison of the relative weights of total visceral fat, total non-visceral fat, and total body fat among the four diet groups is presented in Table 2. There were no substantial differences in the levels of epididymal and perirenal fats. However, a significant (*p* < 0.05) decline in the mesenteric fat in the HF2X group (2.31 g/100 g) compared to the HF group (2.97 g/100 g) was observed. In summary, the total visceral fat accumulation in rats from the HF (11.25 g/100 g), HF1X (9.84 g/100 g), and HF2X (9.29 g/100 g) groups were significantly (*p* < 0.05) higher than those fed the NC diet (4.75 g/100 g). Again, this indicated that the high fat diet was capable of inducing obesity in rats effectively. A significant (*p* < 0.05) reduction in total visceral fat in HF2X group (−17.4%) was noted against the HF group. As for the non-visceral fat levels, an incorporation of the FOS-RMD mixture in high fat diet at different doses also led to significant (*p* < 0.05) reductions in both the HF1X (−17.5%) and HF2X (−20.3%) groups.

Besides this, total body fat was presented as a sum of total visceral fat and total non-visceral fat. Opposing the HF group (67.65 g/100 g) that had the highest body fat, the feeding of FOS-RMD mixture could effectively (*p* < 0.05) lower the total body fat in HF1X (23.76 g/100 g) and HF2X (22.72 g/100 g) diets by −15.5% and −19.2%, respectively. Other authors have also revealed the potential of FOS and RMD in hurdling intestinal fat absorption and mitigating the fat tissue accumulation even though these dietary fibers were administered separately [16]. In this study, an administration of either 2% or 5% of FOS-RMD mixture has contributed to a reduction of fat accumulation in the rats fed different high fat diets.

It was reported that RMD might disturb the uptake of lipid and promote lipid excretion by inhibiting the decomposition of micelles as well as the release of fatty acids from micelles in the small intestine [17]. FOS could directly inhibit the absorption of non-esterified fatty acids or monoglycerides in the small intestine [18]. These findings reinforced our observations in the present study regarding the ability of both the RMD and FOS on alleviating body fat accumulation, at least in part through the mechanism of lowering the dietary lipid absorption and metabolism.

According to Table 3, fecal weight in the NC group was notably (*p* < 0.05) higher than those of the three high fat diet groups, among which no apparent fecal weight changes were observed. This could be possibly attributed to the fact that the NC group had the most total food intake. A mild ascending trend in fecal fat excretion was noted among the three high fat groups on the order of HF (159.5 mg/day) < HF1X (165.0 mg/day) < HF2X (174.5 mg/day). As previously reported by Jun et al. [19], there was a positive relationship between reduced dietary fat absorption and elevated fecal fat output. 

Table 4 shows the changes in the bacterial counts of *Escherichia coli*, *Clostridium perfringens*, and *Bifidobacterium* spp. in different fecal samples. There were no apparent changes in the fecal *E. coli* count (4.73–5.99 log CFU/g) among the four diet groups. *C. perfringens* is known to be harmful by producing toxins that are active in the human gastrointestinal tract [20]. A significant (*p* < 0.05) increase in the fecal *C. perfringens* counts in HF group (5.75 log CFU/g) was observed, showing that high fat consumption might induce a higher growth of *C. perfringens* in the large intestine.

High dietary fat consumption was also found to retard the growth of the *Bifidobacterium* spp. and result in a decrease of this desirable intestinal strain by −23.8% (Table 4). The provision of a FOS-RMD mixture at a high dose could in turn enhance the growth of *Bifidobacterium* spp. by increasing the count from 6.20 log CFU/g (HF) to 8.33 log CFU/g (HF2X). FOS is an indigestible oligosaccharide or dietary fiber that might improve the host health by stimulating the growth or activity of large bowel bacteria, such as *Bifidobacterium* and *Lactobacillus* spp. [9]. To sum up briefly, our results revealed the potential of the FOS-RMD mixture in lowering body fat accumulation and meanwhile improving the growth of intestinal *Bifidobacterium* spp. effectively.

Table 5 presents the SCFA analyses of fecal samples collected from the animals fed the four different diets. High fat consumption resulted in a drastic (*p* < 0.05) reduction of total SCFAs and acetic acid in the fecal samples from 164.1 and 99.1 μmol/g, respectively (NC) to 132.4 and 74.8 μmol/g, respectively (HF). The inclusion of FOS-RMD mixture in the high fat dietary formula, especially at a high dose (HF2X), effectively (*p* < 0.05) elevated the levels of acetic acid, propionic acid, and butyric acid to 152.0 μmol/g (203.2%), 55.9 μmol/g (182.1%), and 50.8 μmol/g (188.8%), respectively, hence increasing (*p* < 0.05) the total SCFA level up to 258.7 μmol/g (195.4%).

In agreement with the findings from Jakobsdottir et al. [21], high fat consumption could cause a reduction in the intestinal total SCFAs. It was inferred that both the FOS and RMD, which were fermented mainly by the increased *Bifidobacterium* spp. count (as shown in Table 4), would give rise to the production of SCFAs, such as acetic acid, propionic acid, butyric acid, and other SCFAs [22,23]. These SCFAs were the key metabolites that reflected the fundamental roles of dietary fibers and gut microbiota in enhancing the intestinal health [24]. To encapsulate the results in both Table 4 and Table 5, the counts of intestinal harmful and beneficial bacteria would decline and escalate, respectively, as the amount of sample intake increased.

## 3. Materials and Methods

### 3.1. Experimental Design and Animal Models

A total of 32 Sprague–Dawley male rats (6 weeks old) weighing 185.4 ± 11.6 g were purchased from the Animal Department of BioLASCO, Taipei, Taiwan. The animal study protocol was approved by the Animal Care and Use Committee of National Chung Hsing University, and the laboratory animals were cared in accordance with the institutional ethical guidelines (IACUC approval number 105-110). The rats were individually housed in stainless steel screen-bottomed cages and placed in a room maintained at 22 ± 1 °C with a 12 h dark/12 h light cycle.

After 7 days of adaptation, the animals were divided into eight weight classes of four each. The rats in each weight class were randomly assigned to one of the four diet groups, including one normal control (NC), one high fat (HF) control, and two high fat experimental diet groups. High-fat diets containing 23% of fat (*w*/*w*) with condensed milk were used to induce obesity [25]. The compositions of the NC and HF diets were shown in Table 6.

In more detail, the two high fat experimental diet groups included one low-dose (HF1X) and one high-dose (HF2X) groups in which FOS and RMD obtained from Syngen Biotech Co., Ltd. (Tainan, Taiwan) were added. Animals in the HF1X and HF2X groups were given a mixture of FOS and RMD (namely FOS-RMD mixture), at a ratio of 1:2. In the HF1X and HF2X groups, animals were given FOS at the doses of 0.48 and 0.97 g/kg body weight, respectively, while the dosages of RMD in these two diets were 0.97 and 1.94 g/kg body weight, respectively.

The animals were fed the four diets for a period of 8 weeks. Throughout the experiment, water and feed were provided ad libitum. Food intakes and body weights were recorded daily. Feces were collected during the last 3 days and stored at −20 °C until analyzed. At the end of experiment, animals were anesthetized by carbon dioxide after fasting for 12 h. After laparotomy, visceral fat pads were excised, blot dried, and weighed. The remaining carcasses were then weighed and stored at −20 °C until analysis.

### 3.2. Feed Efficiency

Feed efficiency for each diet group within the 8-week feeding period was calculated using the following formula:

Feed efficiency = body weight gained (g)/total food intake (g) × 100%.

### 3.3. Measurement of Total Visceral and Non-Visceral Fat

According to the methods of Chu et al. [25], total visceral fat, which was defined as the sum of the mesenteric, epididymal, and perirenal fat depots, were collected and weighted before freezing:

Total visceral fat % = total visceral fat (g)/final body weight (g) × 100%.

In the present study, the non-visceral fat was defined as the total fat in the entire shaved rat torso minus the visceral fat pads. Following the methods of Brooks et al. [26], the carcasses without visceral fat were first autoclaved and then homogenized with water. The homogenized carcass was dried at 65 °C for overnight, followed by drying at 85 °C for another 5 h. Based on the methods of Chu et al. [25], crude carcass fat were extracted from the dried carcass homogenate by ether (1:20, *w*/*v*), and were quantified gravimetrically after evaporating off the solvents in the lipid extracts:

Total non-visceral fat % = crude carcass fat (g)/final body weight (g) × 100%.

In the determination of body fat percentage, total body fat was defined as the sum of total visceral fat and crude carcass fat. Total body fat percentage of each animal was calculated as follows:

Total body fat (%) = [visceral fat (g) + crude carcass fat (g)]/final body weight (g) × 100%.

### 3.4. Determination of Fecal Lipids and Moisture

Crude lipids in the dried fecal samples were extracted with hexane:methanol (2:1, *v*/*v*) mixture based on the procedures described by Folch et al. [27] with slight modifications. Fecal total lipids were quantified gravimetrically after desolventization. Fecal moisture content was determined by drying the fecal sample on aluminum foil trays to a constant weight in an air-oven at the temperature of 105 °C.

### 3.5. Determination of Fecal Short Chain Fatty Acids (SCFAs)

According to the methods described by Huang et al. [28] with slight modifications, fecal samples were first homogenized with cold saline (0.9% *w*/*v*) at a ratio of 1:10 (*w*/*v*). After centrifugation at 1006 *g* for 10 min, 2 mL of the supernatant fraction was acidified with 10 μL of isocaporic acid (internal standard) and 20 μL of 50% (*w*/*v*) sulfuric acid. The SCFAs were then extracted by diethyl ether. After that, 1 μL of the ether layer was analyzed by a column (Agilent J and W HP-INNO Wax GC Column, 30 m, 0.25 mm. 0.25 µm) using a gas chromatograph (Agilent Technologies 7890A, California, CA, USA) fitted with a flame ionization detector. Helium gas was used as the carrier at a flow rate of 7 mL/min. The conditions were as follows: oven temperature, initially held at 80 °C for 1 min and raised to 140 °C at a rate of 20 °C/min, then held at 140 °C for another 1 min and raised again to 220 °C at a rate of 20 °C/min, and lastly held at 220 °C for 2 more min; injector temperature, 140 °C; detector temperature, 250 °C.

### 3.6. Enumeration of Fecal Bacteria

The growth of *Bifidobacterium* spp., *E. coli* and *C. perfringens* were considered as the biomarkers to estimate the intestinal health. After collection, fresh fecal samples were analyzed within 20 min immediately. The fecal samples were immersed and mixed well in a sterile and anaerobic dilution solution (1:10 *w*/*v*), followed by the preparation of serial ten-fold dilutions. *Bifidobacterium* spp., *E. coli* and *C. perfringens* in the solutions were enumerated by different selective and differential medium including iodoacetate medium 25 (BIM-25), Levine eosin methylene blue (LEMB) agar, and tryptose-sulfite-cycloserine (TSC) agar medium (Merck KGaA, Darmstadt, Germany), respectively. These three bacteria were cultivated in an anaerobic incubator at 37 °C for 48 h [29].

### 3.7. Statistical Analysis

All results were expressed in mean ± standard deviation (SD), and analyzed using one-way ANOVA using the SPSS statistics program (version 20.0; SPSS, Armonk, NY, USA). At *p* < 0.05, the differences were considered to be significant.

## 4. Conclusions

Our results indicated that the high fat diets induced fat accumulation in rats and also brought about negative impacts on the intestinal indices, like lesser total SCFA content and higher growth of *C. perfringens*. A supplementation of adequate amount of FOS-RMD mixture (0.97 and 1.94 g/kg body weight) was capable of reducing total body fat, and to counter the above undesirable impacts by stirring up a downward trend in *C. perfringens* counts, but an uphill trend in *Bifidobacterium* spp. counts and SCFA levels.

## Figures and Tables

**Table 1:** A summary of total food intake, body weight, food intake, and food efficiency ratio.

	HF	HF1X	HF2X	Normal Diet
Total food intake (g)	1624.6 ± 39.2*a*	1640.6 ± 27.8*a*	1651.3 ± 59.0*a*	1915.9 ± 75.7*b*
base diet ^1^ (g)	1624.6 ± 39.2*a*	1598.0 ± 28.4*a*	1567.4 ± 59.3*a*	1915.9 ± 75.7*b*
fat content (g)	365.5 ± 8.8	359.5 ± 6.4	352.7 ± 13.3	95.8 ± 3.8
sample given ^2^ (g)	-	42.7 ± 2.1	83.9 ± 5.2	-
Total energy intake (kcal)	6969.5 ± 168.2*a*	6940.8 ± 120.4*a*	6891.9 ± 253.5*a*	6418.3 ± 253.6*b*
from base diet (kcal)	6969.5 ± 168.2*a*	6855.4 ± 121.7*ab*	6724.2 ± 167.7*b*	6418.3 ± 253.6*c*
from sample given (kcal)	-	85.4 ± 4.3	167.7 ± 10.5	-
Initial Weight (g)	245.3 ± 15.2	240.7 ± 11.1	247.3 ± 15.1	239.6 ± 21.1
Final Weight (g)	547.3 ± 33.2	526.2 ± 26.7	509.6 ± 39.4	439.0 ± 37.8
Weight change ^3^ (g/8 weeks)	302.0 ± 28.1*a*	285.5 ± 22.6*ab*	262.3 ± 27.8*b*	199.5 ± 29.7*c*
Feed efficiency ^4^	18.8 ± 1.7*a*	17.4 ± 1.4*ab*	15.8 ± 1.9*b*	10.4 ± 1.6*c*

^1^ Base diet for HF groups: high-fat diet containing 22.5% (*w*/*w*) fat; base diet for Control group: LabDiet 5001. ^2^ Converted from volume fed to rats through gavage. ^3^ Weight change = final weight − initial weight. ^4^ Feed efficiency = [weight change (g)/total feed intake (g)] × 100%. ^5^ Values (means ± SD, *n* = 8) with different letters in the same rows are significantly different (*p* < 0.05).

**Table 2:** Relative size of visceral fat pads and total body fat (g/100 g bw ^1^).

	HF	HF1X	HF2X	Normal Diet
Total visceral fat ^2^	11.25 ± 2.67*a*	9.84 ± 1.24*ab*	9.29 ± 1.26*b*	4.75 ± 0.94*c*
Epididymal fat	3.42 ± 0.71*a*	3.10 ± 0.47*a*	2.89 ± 0.41*a*	1.50 ± 0.29*b*
Mesenteric fat	2.97 ± 0.79*a*	2.59 ± 0.54*ab*	2.31 ± 0.45*b*	1.27 ± 0.27*c*
Perirenal fat	4.85 ± 1.30*a*	4.15 ± 0.58*a*	4.09 ± 0.64*a*	1.98 ± 0.45*b*
Total non-visceral fat ^3^	16.87 ± 3.31*a*	13.92 ± 1.38*b*	13.44 ± 3.14*b*	9.74 ± 2.37*c*
Total body fat ^4^	28.11 ±3.02*a*	23.76 ± 1.84*b*	22.72 ± 3.83*b*	14.50 ± 3.01*c*

^1^ BW = final body weight. ^2^ Total visceral fat % = total visceral fat (g)/final body weight (g) × 100%. ^3^ Total non-visceral fat % = crude carcass fat (g)/final body weight (g) × 100%. ^4^ Total body fat (%) = [visceral fat (g) + crude carcass fat (g)]/final body weight (g) × 100%. ^5^ Values (means ± standard deviation (SD), *n* = 8) with different letters in the same rows are significantly different (*p* < 0.05).

**Table 3:** Fecal weight and fats of the rats fed high-fat diets.

	HF	HF1X	HF2X	Normal Diet
Fecal weight (g/day ^1^)	7.9 ± 1.3*a*	8.9 ± 1.8*a*	8.2 ± 2.7*a*	13.7 ± 0.9*b*
Fecal fat (mg/g feces ^1^)	41.5 ± 7.5*a*	40.7 ± 11.2*ab*	45.1 ± 4.2*a*	26.9 ± 3.0*b*
Fecal fat (mg/day ^1^)	159.5 ± 29.8*a*	165.0 ± 41.9*a*	174.5 ± 40.2*a*	158.9 ± 19.2*a*

^1^ Values (means ± SD, *n* = 8) with different letters in the same rows are significantly different (*p* < 0.05).

**Table 4:** Changes in the fecal *Bifidobacterium* spp., *Clostridium perfringens* and total *Escherichia coli* counts (log CFU/g ^1,2^) among different diet groups.

	HF	1X	2X	Normal Diet
*Bifidobacterium* spp.	6.20 ± 1.11*b*	7.08 ± 1.19*ab*	8.33 ± 1.20*a*	8.14 ± 0.61*a*
*Clostridium perfringens*	5.75 ± 0.47*b*	5.65 ± 0.16*bc*	4.90 ± 1.25*ac*	4.64 ± 0.69*a*
*Escherichia coli*	5.57 ± 1.22	5.99 ± 1.31	4.73 ± 0.75	5.29 ± 0.14

^1^ Bacterial numbers are expressed as log10 colony-forming units per gram of feces. ^2^ Values are presented as means ± SD, *n* = 8 per treatment. Means in a line with no common superscript differ significantly (*p* < 0.05).

**Table 5:** Changes in the fecal short chain fatty acids (μmol/g ^1^) among different diet groups.

	HF	1X	2X	Normal Diet
Total SCFAs	132.4 ± 23.3*b*	221.1 ± 40.7*c*	258.7 ± 19.7*d*	164.1 ± 16.4*a*
Acetic acid	74.8 ± 13.6*b*	120.4 ± 13.7*c*	152.0 ± 13.6*d*	99.1 ± 13.3*a*
Propionic acid	30.7 ± 9.3*a*	43.9 ± 16.9*ab*	55.9 ± 14.8*b*	37.8 ± 4.8*a*
Butyric acid	26.9 ± 6.0*a*	56.7 ± 18.1*b*	50.8 ± 14.5*b*	27.2 ± 3.4*a*

^1^ Values (means ± SD, *n* = 8) in a line with no common superscript differ significantly (*p* < 0.05).

**Table 6:** Compositions of normal chow diet and high-fat diets.

Ingredients	Diet (g/100 g)	
	High Fat Diet	Normal Chow Diet ^1^
Chow	54.0	100.0
Oil	4.8	-
Lard	12.7	-
Condensed milk ^2^	28.5	-
Carbohydrate	41.7	48.7
Fat	22.5	5.0
Protein	15.0	23.9

^1^ Chow (PMI Nutrition International, St Louis, MO, USA) contained crude protein (23.9 g/100 g), crude lipid (5.0 g/100 g), and carbohydrate (48.7 g/100 g). ^2^ Condensed milk contained crude protein (7.3 g/100 g), crude lipid (8.2 g/100 g), and carbohydrate (54.5 g/100 g).
